# Social Organizational Life Cycle Assessment (SO-LCA) and Organization 4.0: An easy-to-implement method

**DOI:** 10.1016/j.mex.2022.101692

**Published:** 2022-04-09

**Authors:** Fernando García-Muiña, María Sonia Medina-Salgado, Rocío González-Sánchez, Irene Huertas-Valdivia, Anna Maria Ferrari, Davide Settembre-Blundo

**Affiliations:** a Department of Business Administration (ADO), Applied Economics II and Fundaments of Economic Analysis, Rey-Juan-Carlos University, Madrid 28032, Spain; b Department of Sciences and Methods for Engineering, University of Modena and Reggio Emilia, Reggio Emilia 42122, Italy; c Gruppo Ceramiche Gresmalt S.p.A, Sassuolo 41049, Italy

**Keywords:** Social sustainability, Social analysis, Stakeholder, Manufacturing, Social value chain

## Abstract

Organizations often face difficulties when measuring their social performance. The lack of international standards, the qualitative/quantitative nature of data, and the unavailability of primary sources all hinder social impact assessments, especially in manufacturing settings. To fill these gaps, the method proposes a simple application protocol of Social Organizational Life Cycle Assessment (SO-LCA), customized for an Italian ceramic tile manufacturer. The method leverages Industry 4.0 digital technologies to collect real-time primary and site-specific social data, making the social assessment dynamic. The managerial approach adopted for the selection of social metrics and weighting of indicators and indexes, can support the transition of the manufacturing organization into Organization 4.0. The method also provides a contribution to the operational validation of the UNEP guidelines by extending their area of application. Finally, the proposed method gives substance to social responsibility through social accounting, helping the organization to measure the correct social impact starting from the detailed data, namely the decisions made in the business and in production.

• Social Organizational Life Cycle Assessment (SO-LCA) application protocol validated in Industry 4.0 environment.
• Social metrics directly linked to production and business processes for the dynamic assessment of social performance.
• Easy replicability of the method in other organizational contexts.

## Graphical abstract

**Figure:**
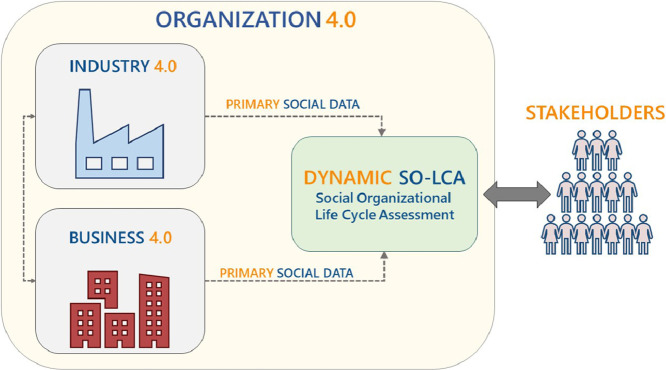

Specifications table

**Table:** 

Subject Area	Economics and Finance
More specific subject area	Social Sustainability Management
Method name	Dynamic Social Organizational Life Cycle Assessment
Name and reference of original method	*Guidelines for Social Life Cycle Assessment of Products and Organizations*. Achten et al. [3] United Nations Environment Programme (UNEP). Paris (2020). p. 138
Resource availability	NA

## SO-LCA method overview

### Methodological background

Companies are increasingly interested in extending the analysis of the environmental impacts of products, services and processes, carried out through Life Cycle Assessment (LCA), to the social dimension. The Social Life Cycle Assessment (S-LCA) and Social Organizational Life Cycle Assessment (SO-LCA) methodologies respond to this need from the perspective of the product and the organization respectively [1]. While LCA is a well-established methodology, regulated by the ISO 14040 series of standards [2], and used in a wide variety of applications, S-LCA/SO-LCA are still in a methodological consolidation phase that follows the United Nations Environmental Programme (UNEP) Guidelines for the Social LCA of Products and Organizations [3].

S-LCA can be understood as a social assessment technique that aims to evaluate the social aspects of products and their potential positive and negative impacts (including potential impacts) throughout their life cycle: extraction, processing of raw materials, production, distribution, use, reuse, maintenance, recycling and disposal [4]. S-LCA can be applied alone or in combination with LCA, but it is important to note that, similar to LCA, it provides guidance on possible improvement actions but cannot determine whether a product is absolutely sustainable [5]. In addition, it is necessary to consider that the viewpoint that S-LCA takes is that of companies wishing to reduce their social impacts, and thus, focuses on the impacts that they can reduce. The most recent literature, while recording numerous contributions of methodological applications and case studies, clearly highlights the difficulty in identifying social indicators at the product level, because social impacts usually occur at the organizational level [6]. Just to solve this critical issue, the UNEP guidelines also provide a general methodological approach for social assessment from an organizational perspective with SO-LCA [7]. However, there are still few studies in the literature that show applications of SO-LCA even in manufacturing. Therefore, the method that is proposed in this paper aims to bridge the aforementioned theoretical gaps by employing the digital technologies of Industry 4.0 as enablers of social assessment in an operational protocol for the effective implementation of SO-LCA [8]. Compared to the UNEP guidelines, the proposed method provides a concrete application example by identifying organization-specific stakeholder and impact subcategories. Thus, this is not only a validation in an operational environment of SO-LCA, but also a methodological extension of the social assessment framework provided in the guidelines.

### Methodological design

The recently updated UNEP guidelines for S-LCA/SO-LCA provide a framework based on the ISO 14040 LCA standards of here follow the same four steps:

(1) Definition of the objective and scope of the analysis;
(2) Compilation of the inventory of inputs and outputs of the system analyzed;
(3) Assessment of the potential impact related to these inputs and outputs;
(4) Interpretation of the results.

In the background, it was highlighted how the assessment of social impacts is still an emerging research area in the manufacturing sector due to the difficulties in identifying social metrics linked to process variables. In order to fill this gap, the proposed method leverages Industry 4.0 digital technologies to automate phase two of the assessment (inventory analysis) by collecting social data in real time [9]. Therefore, the Dynamic SO-LCA method has been validated through the single case study approach [10] considering an Italian company at the forefront of digital transformation and manufacturer of ceramic tiles for building (Gruppo Ceramiche Gresmalt) [11].

## SO-LCA method details

Below are sequentially described all the steps to apply the method in any organizational manufacturing context, consistent with the UNEP guidelines, but extending their application with a deeper level of detail. To this end, six categories and eight subcategories of social impact were identified, along with forty-seven social metrics combined into twenty-eight organization-specific indicators.

### Building the technical-scientific committee

The application of SO-LCA requires the adoption of techniques typical of the social sciences, such as the setting up of a panel of experts of the organization to support the work of the analysts who are responsible for carrying out the social impact assessment. In this application case, 21 top positions have been selected among the board of directors and the top and middle management, as shown in Table 1 [12]. Within the organization analyzed, C-Level managers, while not having operational functions, play a role of major impact because they are directly responsible for the work performed and the results obtained by B-Level managers. Therefore, the composition of the technical-scientific Committee faithfully reflects the organization's decision-making chain. The experts on the committee represent the multiple functional areas of the organization in order to capture different perspectives on social issues.

**Table 1:** Composition of the technical-scientific committee, adapted from Settembre-Blundo et al. [12].

BUSINESS FUNCTION	JOB POSITION
Board of Directors	Chief Executive Officer
Top Management (C-Level)	Chief Financial Officer B2B Sales Director B2C Sales Director Technical Director
Management (B-Level)	Procurement Manager Sourcing Manager Innovation Manager Marketing Manager Administrative Manager Controller Manager HR Manager IT Manager Credit Manager Logistic Manager Security Manager Quality Manager R&D Manager Plant Manager 1 Plant Manager 2 Plant Manager 3

This methodological approach assumes that multiple experts can provide a better evaluation than a single specialist could. The experts are called upon to express their opinions and views. based on their experience and knowledge of the organization, on the subjective choices that social assessment necessarily requires. The analysts conducting the social evaluation have to gather opinions on each methodological issue and, through mutual comparison and progressive sharing, arrive at a holistic synthesis in accordance with social constructivism [13].

### Goal and scope definition

This method aims to describe the implementation of social impact assessment of a manufacturing organization that produces ceramic tiles. This organization is structured in three plants, and one headquarters. The method involves the use of exclusive primary data collected in real time through the digitalization of production and business processes. The reporting organization was chosen as the unit of analysis by setting the boundaries of the system "*from cradle to grave*", in accordance with the UNEP guidelines for SO-LCA [3].

At this stage of the analysis. it is also necessary to identify and categorize stakeholders that are potentially key to the organization. The UNEP guidelines set out six main stakeholder categories (workers, local community, society, consumers, value chain actors and children). with six corresponding impact categories (human rights, working conditions, health and safety, cultural heritage, governance and socio-economic impacts) associated with them. Because this method aims to use organization-specific social metrics and primary data only, the choice of key stakeholders must represent the real value chain. The guidelines allow for customization of stakeholder selection criteria and for defining the most appropriate impact categories for social assessment.

Table 2 shows the framework for categorizing stakeholders and impact categories for Dynamic SO-LCA. The category of children was not included because it was considered irrelevant to the manufacturing dimensions of the value chain of the organization undergoing this social assessment. For the impact categories in this method the four capitals (human, social, natural and economic) are proposed in accordance with the theory of capitals for sustainability [14] and sustainable development [15]. Then, for each impact category, several stakeholder sub-categories associated with additional impact sub-categories were identified. The criterion followed in choosing the sub-categories of stakeholders was that of representing, as fully as possible, the organization's value chain with data coming only from primary sources. Among these, category *3.Society* included subcategory *3.3 Environment*, to provide the method with a direct link to the environmental assessment that could be conducted in parallel with the social one. Finally, the Stakeholder Details shows all those stakeholders who are specific to the organization under analysis and to whom it is possible to relate social metrics to measure impact based on primary data sources.

**Table 2:** Framework for the selection and ranking of Stakeholders and impact categories, adapted from García-Muiña et al. [8].

### Inventory analysis

The first step in inventory analysis is to identify the social metrics through which to conduct the social assessment. They must be specific and characteristic of the organization and must also be directly and easily measurable in order to have the primary data as set by the goal for this model of analysis. To this end, the technical-scientific committee (Table 1) has identified and chosen a series of metrics that meet the methodological requirements set, correlating them with the categories of stakeholders and social impact through a matrix (Table 3).

**Table 3:** Matrix of social metrics categorized with respect to impact and stakeholder categories, adapted from García-Muiña et al. [8].

The stakeholder category *1.Workers* corresponds to the impact *A.Human Capital* and consists of fourteen metrics related to human resources. These include the number of Personal Protective Equipments (PPEs) provided to employees over the time considered (MA1.8) and the number of Collective Bargaining Agreements (CBAs), (MA1.9). The *2.Local Community* category, correlated to the *B.Social Capital* impact, considers both, more generally, the stakeholders selected (MB2.1) and those involved by the organization (MB2.2), and specifically, the local administrations identified (MB2.3) and directly involved in the company's activities (MB2.4). Still linked to the impact of *B.Social Capital* is the category *3.Society,* which includes various metrics, including man-hours dedicated to research, development and innovation (MB3.2) and man-hours worked by scientists from research centers and universities that collaborate with the organization in joint research projects. Then the Regulatory Authorities involved (MB3.4) and those directly involved (MB3.5) are considered. Finally, to measure the effect of social networks on the reputation of the organization, both the number of followers and likes received for posts on corporate and commercial profiles, both business-to-business (B2B) and business-to-consumer (B2C), were included. The environment was introduced as a stakeholder in the *3.Society* category, associating it with the impact category *C.Natural Capital* and adopting as metrics the Global Warming Potential (GWP) of the organization (MC3.1) and that of the sector (MC3.2), *4.Consumers* has been associated with impact category *D.Economic Capital,* considering both non-compliance costs and turnover generated in business-to-business and business-to-consumer channels. Finally, again among the economic metrics (*D.Economic Capital*) characteristic of the *5.Value Chain Actors* category, human resources dedicated to research and innovation, the amount of investment and the way purchase orders are approved were included. Relevance is also attributed to suppliers who are categorized, as key, local and ethical if they have signed the organization's code of ethics.

**Fig. 1:**
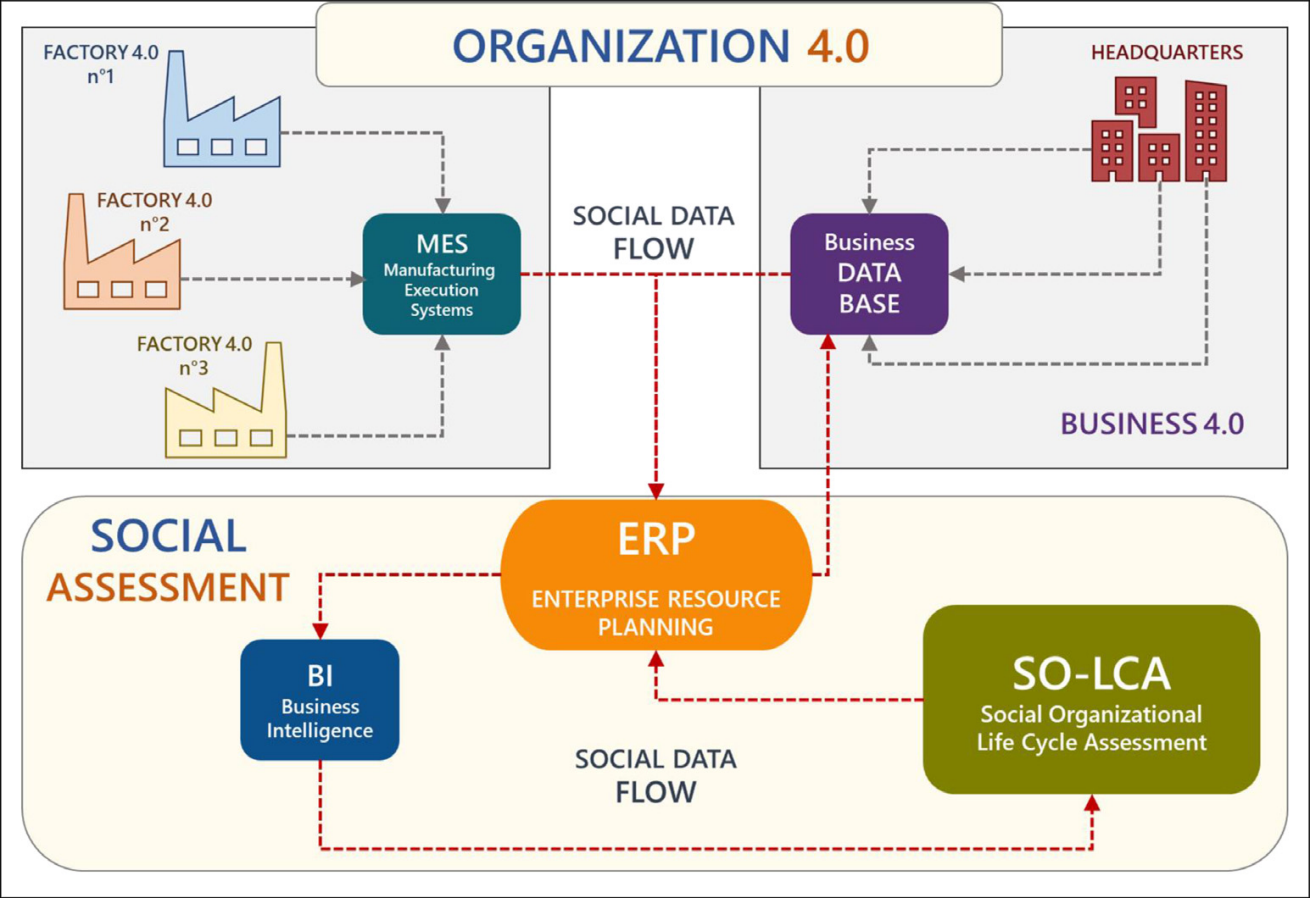
Organization 4.0-based dynamic inventory analysis, adapted from García-Muiña et al. [8].

This application method of SO-LCA is based on Dynamic Inventory Analysis (DIA) which, by exploiting the potential of Industry 4.0 digital technologies, makes the collection of primary social data from the organization an automatic process (Fig. 1). The data for the social impact assessment comes from both the production units (factories) and the headquarters. The technological hub of the method is the ERP (Enterprise Resource Planning) system, it collects both production information coming from the factories through a MES (Manufacturing Execution System) and management information exploiting a Business Data Base which also provides data from the value chain (suppliers. distributors and customers). Finally, a Business Intelligence (BI) system interfaces the ERP with the SO-LCA calculation tool.

Factory 4.0 is an environment in which the physical and digital dimensions are interconnected, enabling multi-directional communication between production processes and products in real time. The MES in Factory 4.0 allows to collect data from different sources and on these to carry out analysis. Business 4.0 is the extension of the Industry 4.0 paradigm to the corporate governance, providing a framework of business behavior that optimizes the digital advantage to create value for stakeholders. Digital technologies therefore enable the collection not only of factory data, but also, those closely related to business processes and representing the organization's value chain: suppliers, customers, human resources, investors, institutions, etc. This information is collected in a Business Data Base which, together with the MES, communicates with the ERP transferring also the data necessary for the social evaluation. As a calculation tool for the SO-LCA was used a Microsoft Excel® multiple spreadsheet which was integrated with the ERP (SAP™) thanks to Microsoft Power BI® used as a business intelligence system. Organization 4.0 is a dynamic configuration that makes social data available in real time thus enabling the social impact assessment as it occurs. This is an innovative approach compared to conventional social assessments that instead rely on data corresponding to conditions and states that have already been experienced.

This Dynamic Inventory Analysis scheme, of course, can be easily applied in those organizations that have already achieved a high rate of digitalization of their processes. In other cases, however, the model can be easily implemented through a more traditional analogical data collection by analyzing specific time periods such as one or more years already passed.

### Social impact assessment

To perform the impact analysis, the 46 social metrics already selected were combined into 28 organization-specific social impact indicators as shown in Table 4. In the SO-LCA methodology, impact assessment is sought through the effects that the organization's activities have on its stakeholders and to this end, indicators were associated with both endpoint impact categories and midpoint impact subcategories [16].

**Table 4:** Description of Social Indicators and their contribution to social sustainability, adapted from García-Muiña et al. [8].

In order to facilitate the association of social metrics and indicators to the different impact categories and sub-categories, an alphanumeric coding was adopted in this method, as shown in Table 4, which allows to trace the construction of indicators and their correspondence with stakeholders. Combining the metrics with each other, as proposed, is intended to have social indicators specific to the manufacturing organization. In addition, to facilitate visual association between indicators and impact categories and subcategories and stakeholder categories and subcategories, four different colors were adopted to identify the four capitals considered (human, social, natural, and economic). Since social metrics contribute in different ways to social sustainability, the measurement of indicators must consider this and, therefore for each of them, it was specified whether the increase or decrease in their value corresponds to a positive or negative social effect.

### Method validation

To validate the method, a series of dynamic data collections were performed (as described in Fig. 1) on a daily basis during the month of November 2021, which was a period of full activity for the organization and therefore representative for the social assessment. This screening showed that, under stable operating conditions, the value assumed by the social metrics are constant, so to calculate the social indicators in Table 4, their values were averaged. The absolute values of the social metrics are not reported because they are sensitive data for the organization under analysis. Instead. the values taken by the social indicators are shown in the form of a metrics ratio, as shown in Table 5.

**Table 5:** Evaluation and rating of Social Indicators, adapted from García-Muiña et al. [8].

Since the indicators, depending on the case, can describe a positive social contribution either when they take on a high or low value, the method provides for their standardization to facilitate their reading and analysis. In accordance with the UNEP guidelines for S-LCA [3] and the literature [17], the indicators were classified by adopting a 5-point likert scale [18,19] as shown again in Table 5:

(1) Value 0.2 - starkly below compliance level;
(2) Value 0.4 - slightly below compliance level;
(3) Value 0.6 - compliance with local and international laws and/or basic societal expectations;
(4) Value 0.8 - beyond compliance;
(5) Value 1.0 - ideal performance. best in class.

The technical-scientific committee experts (Table 1) associated the value expressed by the social indicators with the 5 levels of the above rating by employing the following value ranges.

Rating value (range of value assumed by the social index);

• 0.2 (0.0 ÷ 0.2);
• 0.4 (0.2 ÷ 0.4);
• 0.6 (0.4 ÷ 0.6);
• 0.8 (0.6 ÷ 0.8);
• 1.0 (0.8 ÷ 1.0).

However, not all indicators were able to have the best performance associated with rating 1, and these are the exceptions:

• 0.5 (Gender Parity);
• >0.1 (Migrant worker);
• 0.05 (Training);
• 0.1 (R&D workforce and innovation workforce).

Finally, for the child labor and forced labor indicators, only the best social performance corresponding to rating 1 is obviously allowed.

Each expert's attribution of the rating value for each social indicator was collected and the set of values obtained was then integrated by applying the following formula (1):

(1) $${\left(r\right)}_{i}=\;\frac{\sum_{i=1}^{n}{\left(e\right)}_{i}}{n}$$

In the formula *(r)_i_* is the integrated value of the social indicator *i* obtained by summing the rating values attributed by the experts for the same indicator *(e)_i_*, while *n* is the number of experts who make up the committee (in this case 21). This provides a framework of normalized and comparable social indicators (Table 5).

The next step in impact assessment is to integrate the different social indicators to build social indexes corresponding to the different subcategories and categories of social impact. In this method, integration is carried out through weighted aggregation of the indicators [20]. In this way, it is possible to overcome the limitation of assigning equivalent weights in the weighting for S-LCA studies, as highlighted in the literature in cases of lack of primary data. Since this method relies on the use of primary data and specific metrics, the committee experts were asked to assign a weight (expressed in%) for each indicator in order to achieve a weighted aggregation by applying the following formula (2):

(2) $${\left(s\right)}_{i}=\sum_{i=1}^{m}{\left(r\right)}_{i}\times w_{i}$$

In the formula, *(s)_i_* is the aggregate Social Index by subcategory or impact category *i*, while *(r)_i_* are the social indicators, *w_i_* is the weight (%) given by the committee experts, and finally *m* is the number of subcategories or impact categories.

The operations described above that determine the social rating values shown in Table 5 are intended to standardize the indicators and make it easier to read and compare them with each other. Otherwise, there would be no comprehensive view of the organization's social performance.

Table 6 (a) shows the complete picture of the Social Indexes attributed for each subcategory and impact category correlated with the corresponding subcategories and stakeholder categories. With a simple mathematical average of the endpoint indexes, it is also possible to obtain the Total Social Index. In order to verify the degree of decision uncertainty the same expert panel was asked to give an assessment of the weighting of the indicators six months after the first analysis. Table 6 (b) shows that the new results are quite similar to those obtained previously, thus demonstrating the reproducibility of the scores. Clearly, this framework reflects the organizational perspective of the experts on the technical-scientific committee. However, beyond the absolute results obtained in this case, which served to demonstrate the easy application of the method, the weights and weighting criteria can be easily tailored to the different contexts in which this method may be replicated. For example, to neutralize the potential conflict of interest of the panel of experts, all of whom belong to the same organization, the choice of weights for weighting could be made by engaging some of the key stakeholders for each impact category who could join the Committee by providing an interpretive perspective from outside the organization.

**Table 6:** Evaluation and rating of Social Indexes, adapted from García-Muiña et al. [8].

**(a)**
**(b)**

In order to facilitate the validation of the method, the values assumed by the different sustainability indices (Table 6) were configured to build the value chain in which the organization operates, aligning the different subcategories of impact in the perspective of Life Cycle Thinking [21].

**Fig. 2:**
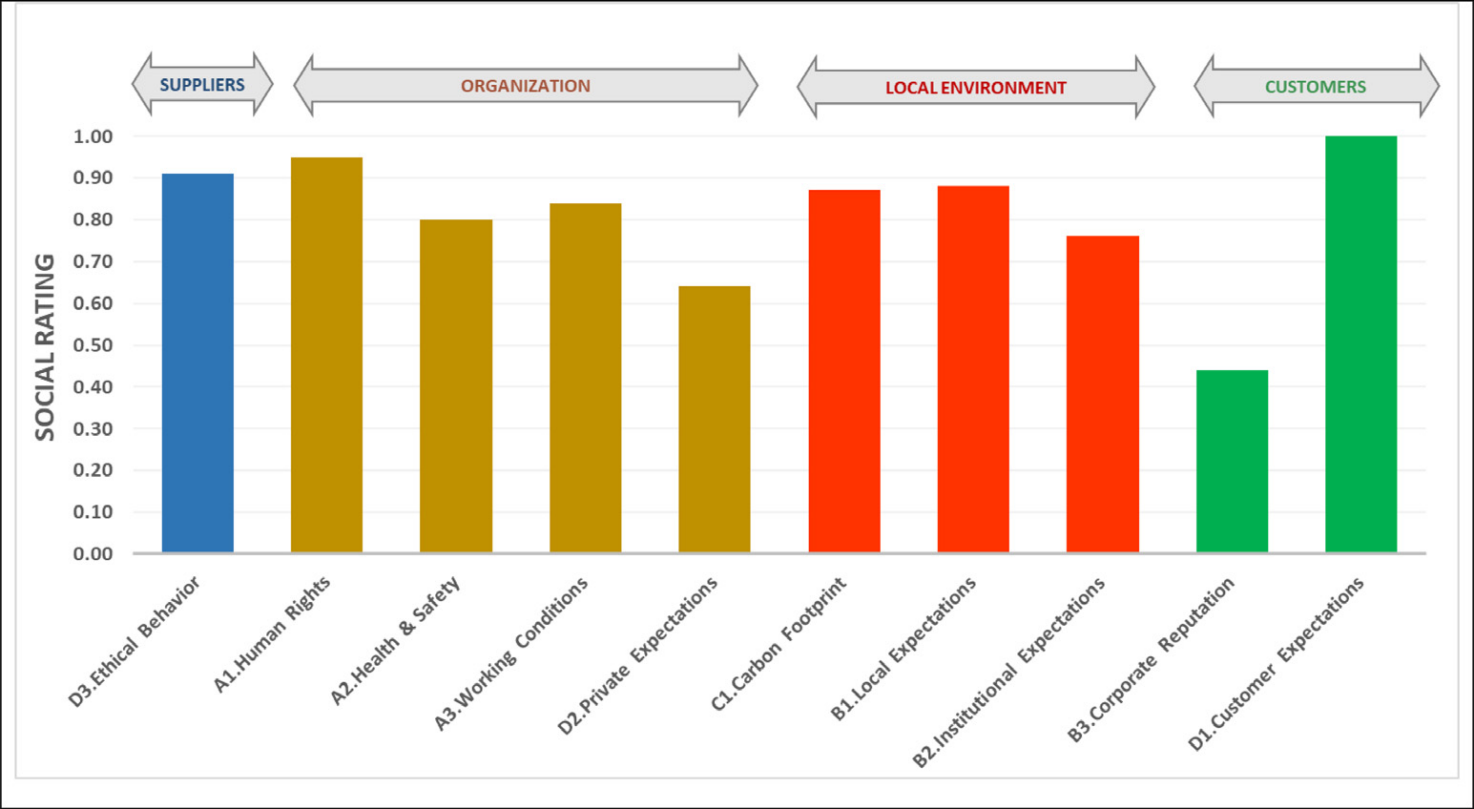
Trend of social impact along the organization's value chain, adapted from García-Muiña et al. [8].

In Fig. 2, the social impacts are represented by the values assumed by the midpoint indices for each subcategory of social impact. Therefore, in the case of the organization that was analyzed to show the effectiveness of the method, it appears that the social performance is excellent (almost all of them close to the maximum value 1), moreover, areas of possible improvement also emerge, namely Private Expectations and Corporate Reputation.

## Conclusion

Considering the need to effectively implement the SO-LCA to support sustainability decision-makers [7], this method proposes an implementation protocol for Social Organizational Life Cycle Assessment (SO-LCA) based on the guidelines provided by UNEP and tested in a manufacturing environment. The approach followed by the proposed method served to fill the main gap highlighted in the literature, namely the lack of primary, site-specific social data that forces the use of general secondary data sources. The method solves these critical aspects by identifying a set of social metrics native to the analyzed organization that, combined into indicators and indices, provide the extent of social impact with respect to different categories and subcategories of stakeholders. The method also demonstrates the great potential of Industry 4.0 technologies to enable social assessment. The inventory analysis was conducted by querying the organization's ERP, which also made primary social data available in real time for a dynamic social impact assessment. It follows that the Industry 4.0 operating model, specific to the factory environment, can support the transition from a traditional organization to a data-driven Organization 4.0. On the other hand, in the case of organizations with a lower level of digitization of their operations than expected by the Industry 4.0 paradigm, the method can be equally successfully applied. Table 7 schematically shows the difference between the two approaches: the digitization of the organization enables the dynamic, real-time collection of primary data while also supporting its processing; in a still analog operating environment, data collection is instead conducted manually using business information from past periods, or from secondary sources.

**Table 7:** Steps in the social assessment of organizations with different digital maturity.

N°	SO-LCA STEPS	ORGANIZATION 4.0	ORGANIZATION 2.0
**1**	**Technical-scientific committee constitution.**	Executed by the decision makers supported by the analyst	Executed by the decision makers supported by the analyst
**2**	**Goal and scope definition.**	Performed by the analyst and driven by the data	Performed by the analyst
**3**	**Social inventory analysis.**	Dynamic and real-time data collection	Static and retrospective data collection
**4**	**Social impact assessment.**	Automatic processing	Manual processing
**5**	**Results Interpretation.**	Supported by business intelligence	Performed by the analyst

The proposed method can help organizations to analyze their social variables, monitor their performance, identify responsibilities, areas and ways of improvement. Moreover, the data obtained, in a perspective of accountability, can be used to communicate and dialogue with stakeholders in a more transparent way, considering their expectations in decision-making processes.

## Acronyms

• **BI:** Business Intelligence
• **B-level:** mid-level managers
• **CBAs:** Collective Bargaining Agreements
• **C-level:** executive-level managers
• **ERP:** Enterprise Resource Planning
• **LCA:** Life Cycle Assessment
• **MES:** Manufacturing Execution System
• **PPEs:** Personal Protective Equipments
• **S-LCA:** Social Life Cycle Assessment
• **SO-LCA:** Social Organizational Life Cycle Assessment
• **UNEP:** United Nations Environmental Programme

## CRediT authorship contribution statement

**Fernando García-Muiña:** Investigation, Writing – original draft. **María Sonia Medina-Salgado:** Methodology, Project administration. **Rocío González-Sánchez:** Formal analysis, Validation. **Irene Huertas-Valdivia:** Resources. **Anna Maria Ferrari:** Supervision. **Davide Settembre-Blundo:** Conceptualization, Writing – original draft.

## Declaration of Competing Interest

The authors declare that they have no known competing financial interests or personal relationships that could have appeared to influence the work reported in this paper.
